# Efficacy and Safety of Tranexamic Acid in Shoulder Arthroscopic Surgery: A Systematic Review and Meta-Analysis

**DOI:** 10.3390/jcm11236886

**Published:** 2022-11-22

**Authors:** Yiyuan Sun, Dan Xiao, Weili Fu, Wufeng Cai, Xihao Huang, Qi Li, Jian Li

**Affiliations:** 1 Day Surgery Center, West China Hospital of Sichuan University, Chengdu 610041, China; 2 Yong Chuan Hospital of ChongQing Medical University, Chongqing 402160, China; 3 Department of Orthopedics, Orthopedic Research Institute, West China Hospital of Sichuan University, Chengdu 610041, China

**Keywords:** tranexamic acid, arthroscopy, shoulder, rotator cuff repair, hemarthrosis

## Abstract

Background: Visual clarity during shoulder arthroscopy can ensure an efficient and effective performance of the procedure, and it is highly related to bleeding without a tourniquet. Tranexamic acid (TXA) is widely used in adult reconstruction procedures; however, its use in shoulder arthroscopic operations is a relatively novel topic. Purpose: To analyze the available literature on visual clarity, blood loss, pain control, functional outcomes, and complications after the administration of tranexamic acid in shoulder arthroscopic surgery. Methods: A literature search was performed to retrieve randomized controlled trials examining the use of tranexamic acid at the time of shoulder arthroscopic surgery. The literature search included the MEDLINE, Embase, Web of Science, and Cochrane Library databases. The primary outcomes included visual clarity, blood loss, and visual analog scale scores for pain. Secondary outcomes were operative time, irrigation amount used, postoperative shoulder swelling, the need for pressure increase, mean arterial pressure (MAP), functional outcomes, postoperative adverse effects such as deep venous thrombosis, and pulmonary embolism. The outcomes were pooled to perform a meta-analysis. Results: Seven prospective randomized controlled trials met the inclusion criteria for analysis. All of the included studies performed arthroscopic rotator cuff repair. No significant difference in visual clarity was observed (SMD (standardized mean difference), 0.45 [95% CI(confidence interval), −0.68, 1.59]; *p* = 0.44) nor in pain score (MD (mean difference), −0.46 [95% CI, −0.97, 0.05]; *p* = 0.08) between the TXA group and the control group. Two studies found no significant difference in blood loss between the TXA group and the control group. The meta-analysis from five studies demonstrated no significant difference between the TXA and control groups in operative time (MD, −3.51 [95% CI, −15.82, 8.80]; *p* = 0.58) or irrigation amount used (MD, −2.53 [95% CI, −5.93, 0.87]; *p* = 0.14). Two trials reported different statistical results in postoperative shoulder swelling. No significant differences regarding the need for pressure increase and MAP were reported between groups. No wound complications or infections or cardiac, thrombotic, or thromboembolic complications were recorded in either group. Conclusion: The use of intravenous or local TXA in shoulder arthroscopic surgery did not increase complications or thromboembolic events, but TXA had no obviously effect of reducing bleeding to obtain a clear visual field or pain release in patients undergoing shoulder arthroscopic surgery.

## 1. Introduction

Shoulder arthroscopic surgery requires soft tissue release and bone preparation, resulting in bleeding, hematoma formation, and postoperative pain [1]. Adequate visualization, which is highly related to bleeding during arthroscopy, is of great importance [2]. Unlike lower extremity surgery, tourniquets cannot be used for shoulder arthroscopic surgery. In comparison, the methods of reducing intraoperative bleeding mainly rely on controlled blood pressure reduction and plasma radiofrequency hemostasis, and a large amount of normal saline irrigation is required during the operation [3,4]. Swelling of the shoulder joint, more wound exudation, and subcutaneous blood stasis often occur after surgery. Tranexamic acid (TXA) is a synthetic derivative of lysine and works as an antifibrinolytic owing to its ability to reversibly block the activation of plasminogen [5]. TXA is commonly used in upper and lower limb arthroplasty to limit blood loss and postoperative hematoma formation [6,7]. Tranexamic acid has been evaluated for its usefulness in reducing blood volume loss, drain output, and hematocrit changes after shoulder arthroplasty [8]. Additionally, TXA has been shown to decrease blood loss, drain output, and hemarthrosis, and improve pain scores and range of motion in the initial postoperative period, without increased complications or thromboembolic events after anterior cruciate ligament reconstruction and the open Latarjet procedure [9,10]. However, there is limited evidence and small sample sizes to support its use in shoulder arthroscopic surgery.

A recent randomized controlled trial by Liu et al. [11] assessed the ability of TXA to improve visualization during arthroscopic rotator cuff repair (RCR). They demonstrated improved visualization and early postoperative pain scores. The same effect was concluded in other reports [12,13]. However, for the image quality of the individual stages of the operations, the difference found was not statistically significant [12]. Additionally, Mackenzie et al. [14] and Takahashi et al. [15] concluded that TXA did not improve postoperative pain scores after RCR in a double-blind, randomized controlled trial.

A meta-analysis of the data from different studies may facilitate clinical decision-making regarding the use of TXA, including support for the accurate assessment of some rare complications and their clinical significance. The aim of this systematic review and meta-analysis was to investigate the clinical usefulness and effectiveness of TXA in shoulder arthroscopic surgery. Our hypothesis is that TXA can reduce bleeding effectively so as to obtain a clear visual field and pain release in patients undergoing shoulder arthroscopic surgery.

## 2. Methods

### 2.1. Search Strategy

This systematic review and meta-analysis was performed in accordance with the Preferred Reporting Items for Systematic Reviews and Meta-Analysis (PRISMA) statement using the Cochrane Review methods [16]. The PROSPERO database was searched, which revealed no ongoing or recently completed systematic reviews on this topic, and we applied a registration number (CRD42022352420). MEDLINE, Embase, Web of Science, and the Cochrane Library were searched from their inception to 10 August 2022. The references of the potentially relevant included studies were checked to enhance the recall ratio. The key words used in the search included “tranexamic acid”, “TXA”, ”shoulder”, “arthroscopy”, and “rotator cuff”. The literature search was repeated before submission, on 26 August 2022, to ensure that no additional studies had been published since the original search was performed.

### 2.2. Inclusion and Exclusion Criteria

Studies were included if they met the following criteria: (1) the studies were human subjects and RCTs (randomized controlled trial) published at any time; (2) the primary outcomes were at least one of visual clarity, pain score, or blood loss; (3) one of the intervention groups was that the patients received TXA without restrictions on dosage and the route of administration; and (4) the full text was available without language restriction. The exclusion criteria were papers that did not perform shoulder arthroscopy. Two authors (D-X and YY-S) independently checked the studies identified from the searches against the inclusion criteria. The third author (WF-C) arbitrated in cases of any difference.

### 2.3. Quality Appraisal and Assessment of Risk of Bias

The quality and assessment of risk of bias for each study included in the review and meta-analysis were independently rated by the two authors (XH-H and WF-C). After that, the senior author (WL-F) verified with the Cochrane Collaboration tool for RCTs, which evaluates the risk of the following biases: reporter (selective reporting), attrition (incomplete outcome data), detection (blinding of outcome assessment), performance (blinding of participants and personnel), selection (random sequence, allocation concealment), and others. Individual bias was classified as being of low (score 4–7, high quality) or high (score 1–3, low quality), according to the Modified Jadad Scale.

### 2.4. Data Collection and Abstraction

Two reviewers (D-X and YY-S) independently extracted data from the studies involved and recorded the items in the data extraction form, which was tested before the formal extraction process. The items were as follows: the author, publication year, country, level of evidence, sample size, surgical procedure, anesthesia measured, route of TXA administration and dose, intervention of the control groups, postoperative rehabilitation, follow-up, and complications. The primary outcomes were visual clarity in the operation, pain score, and blood loss. Secondary outcomes included operative time, length of stay, swelling, analgesic usage, irrigation amount used, thromboembolic events, wound complications, and mortality. Differences between the two reviewers were resolved by discussion with the third reviewer (WF-C).

### 2.5. Meta-Analysis Methods and Subgroup Analysis

The meta-analysis was conducted when sufficient data existed from ≥3 studies. Data analysis was performed using Review Manager (RevMan Version 5.3; Nordic Cochrane Centre; Copenhagen, Denmark). The heterogeneity of the included RCTs was assessed via the chi-square test and I^2^ statistic. For the χ^2^ test, *p* < 0.05 indicated significant heterogeneity. In accordance with the Cochrane Handbook for Systematic Reviews of Interventions, we used the following method for interpreting I^2^: 0% to 40% might not be important; 30% to 60% may represent moderate heterogeneity; 50% to 90% represent substantial heterogeneity; 75% to 100% represent considerable heterogeneity [17]. I^2^ < 50%, *p* ≥ 0.1, indicated that there was no statistical evidence of heterogeneity, and a fixed-effects model was adopted; otherwise, a random-effect model was used. *p* values less than the alpha level, which was set at 0.05, were considered statistically significant. If data were available, the authors planned to perform a subgroup analysis including different routes or dosages of TXA administration. A sensitivity analysis was conducted to explore potential explanations for the high heterogeneity. Potential publication bias was assessed by using a funnel plot when a single outcome existed from ≥8 studies.

## 3. Results

### 3.1. Study Selection

**Figure 1:**
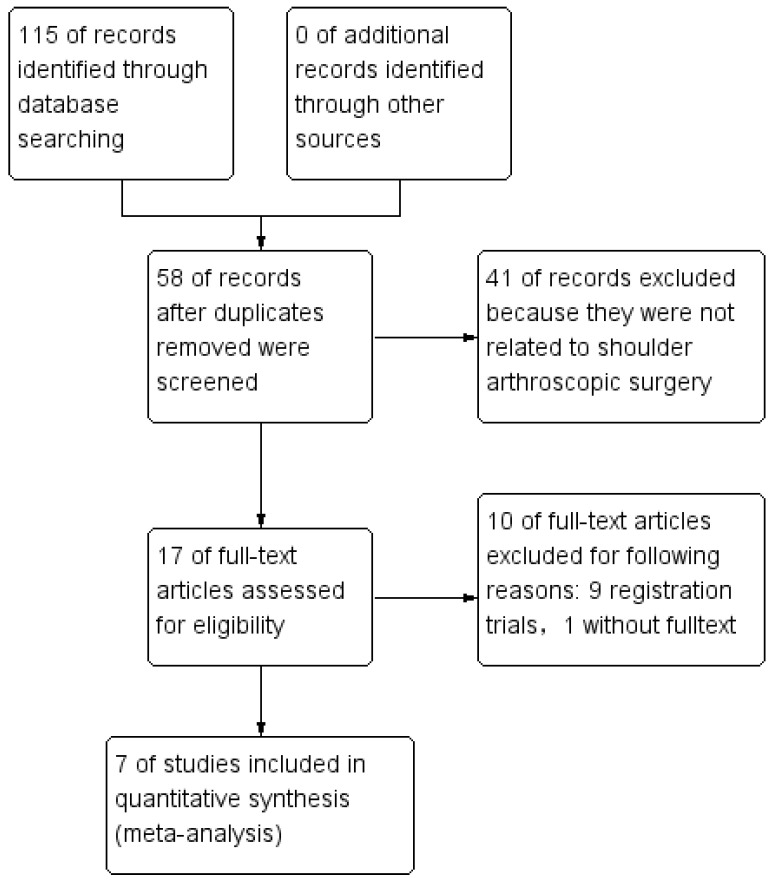
Study flow diagram.

The details of the study selection process are presented in Figure 1. The literature search of the MEDLINE (17), Embase (25), Web of Science (18), and Cochrane Library databases (55) identified 115 RCTs. No additional studies were identified from the references of the included studies. Fifty-seven RCTs were excluded because of duplicate studies, and 41 RCTs were excluded after the titles and abstracts were scanned because they were not related to shoulder arthroscopic surgery. Registration trials without results were posted for nine studies. The full text was not found in one article. We finally included the following seven studies in this meta-analysis: Takahashi et al. [15], Bayram et al. [18] Mackenzie et al. [14], Liu et al. [11], Ersin et al. [12], Gao et al. [19], and Nicholson et al. [20].

### 3.2. Search Results

Seven studies met the inclusion and exclusion criteria, including 272 patients undergoing shoulder arthroscopy with TXA and 265 patients without TXA (Table 1). The trials were published between 2019 and 2022. Of these, two were conducted in China, two in Turkey, one in the United States, one in Japan, and one in Australia. Of these included RCTs, three were level I evidence [14,15,18] and four were level II evidence [11,12,19,20]. All of the included studies comprised patients undergoing arthroscopic rotator cuff repair. Six trials used general anesthesia [11,12,14,15,19]. One of them was combined with an echo-guided interscalene plexus block [14]. Another study performed a preoperative interscalene brachial plexus nerve block [20]. Five studies compared TXA intervention against saline [11,12,14,15,19]. One study compared TXA with epinephrine [18]. The control group underwent rotator cuff repair without TXA in another trial [20]. TXA (1 g to 2 g) was administered intravenously before surgery in four studies [11,14,15,20]. The procedure in another three studies was performed through the bolus IV administration of 10 mg/kg TXA in 100 mL isotonic saline solution, intraarticularly by injection into the shoulder joint and subacromial space or using irrigation fluid containing 0.42 mg of TXA per 1 L of saline [12,18,19,21]. Postoperative rehabilitation was described in four studies [11,14,15,19], and the treatment of pain control was reported in two studies [11,15]. Complications were limited to six cases of secondary adhesive capsulitis and threecases of re-tears [14]. Additionally, six cases in the TXA group and two cases in the placebo group experienced subcutaneous bloody ecchymosis [19]. Four articles described the duration of follow-up from 1 week to 52 weeks [11,14,15,19]. No side effects, such as thromboembolic effects, wound complications, or infections, were noted in any of the included studies.

### 3.3. Quality Appraisal and Risk of Bias

**Figure 2:**
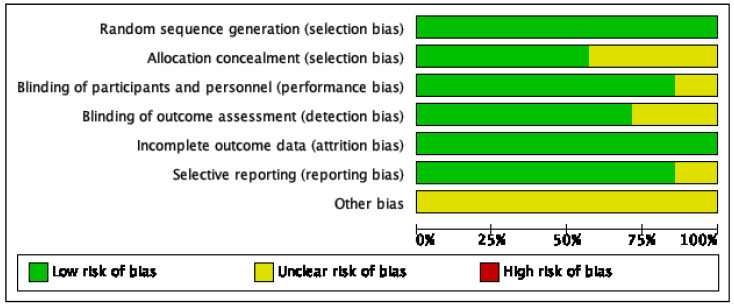
Risk of bias graph: review authors’ judgments about each risk of bias item presented as percentages across all of the included studies.

**Figure 3:**
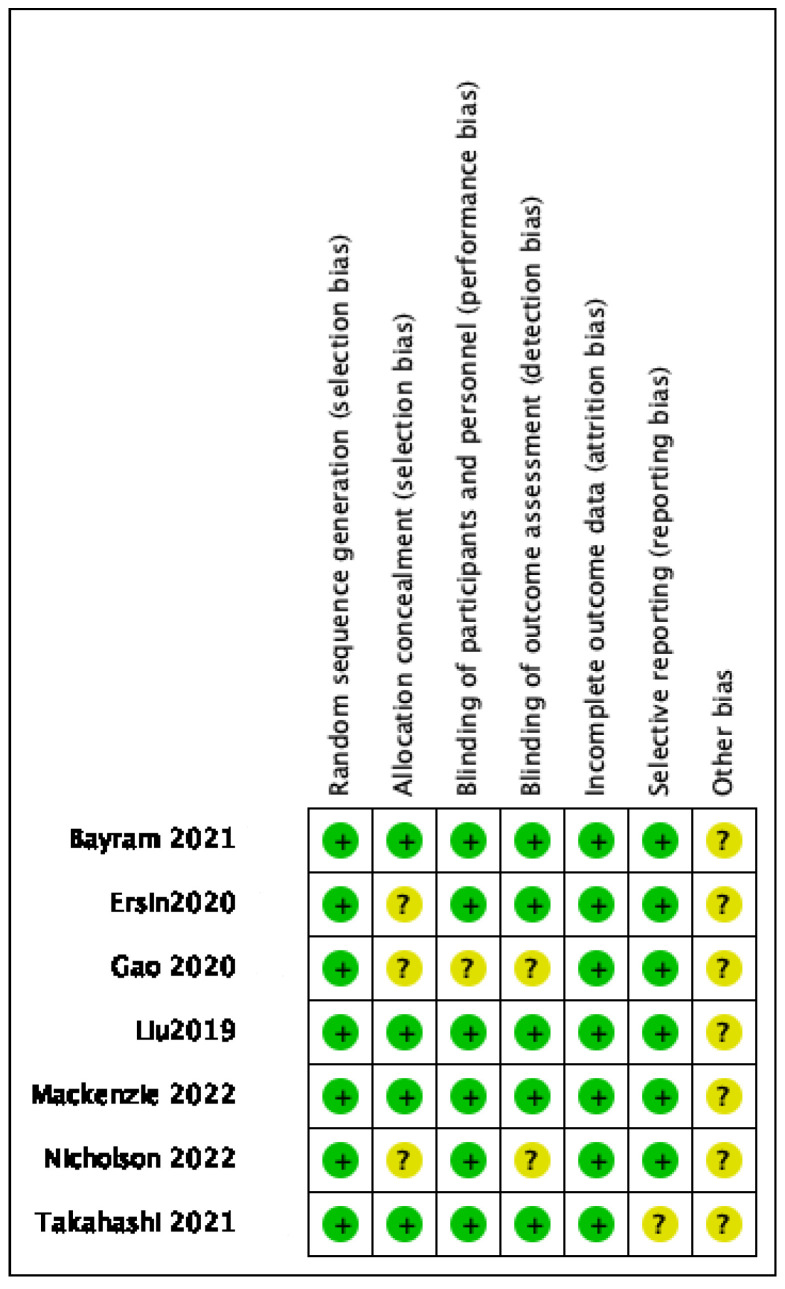
Risk of bias summary: review of authors’ judgments about each risk of bias item for each included study [11,12,14,15,18,19,20].

The risk of bias of the included studies is summarized in Figure 2 and Figure 3. All of the studies were assessed as being high quality according to the Modified Jadad Scale at the same time (Table 2). Sequence generation was adequately reported by all of the studies (low risk of bias). Four studies described allocation concealment [11,12,14,18]. In six studies, patients and surgeons were blinded to treatment allocation (low risk of bias), while one study did not report whether patients and/or surgeons were blinded to treatment allocation (unclear risk of bias) [19]. Blinding of the outcome assessor was not mentioned in two studies (unclear risk of bias) [19,20], and the absence of follow-up was reported in two studies, but the number of and reason for missing people were similar (low risk of bias) [14,18]. It was clear that all of the prespecified and expected outcomes of interest were reported in all studies. Published bias could not be conducted for only seven studies, but was increased by a trial with only the abstract and by nine ongoing trials [13,22,23,24,25,26,27,28,29,30].

### 3.4. Primary Outcomes

#### Visual Clarity

**Figure 4:**
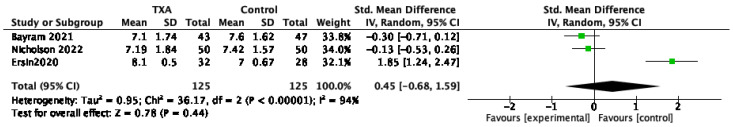
Forest plot and meta-analysis of visual clarity.XA groups. The centre of each square represents the weighted mean differences for individual trials, and the corresponding horizontal line stands for a 95% confidence interval. The diamonds represent pooled results. Legend: CI = confidence interval; SD = standard deviation [12,18,20].

Five included studies measured visual clarity during the operation [11,12,15,18,20]. Three studies [12,18,20] used a 10-point visualization scale; 0 was categorized as “poor” and 10 was categorized as “good”. The meta-analysis from these three studies demonstrated no significant difference in visual clarity score between the TXA and control groups (SMD, 0.45 [95% CI, −0.68, 1.59]; *p* = 0.44; Figure 4). Statistical assessment for heterogeneity found for the VAS scores of the TXA versus control groups was I^2^ = 94% (*p* < 0.00001). A random-effect model for high heterogeneity and SMD (mean difference) was applied because different calculation methods were used. The large degree of statistical heterogeneity indicates underlying differences among the studies. As we described previously, arthroscopy in Bayram’s study was performed using irrigation fluid containing 0.33 mg of epinephrine per 1 L of saline in the control group and 0.42 mg of TXA per 1 L of saline in the TXA group. There was no significant difference in the surgeon-rated visual clarity between the groups, which means there was a similar visual quality between groups. Two studies used a three-grade visual clarity scoring system to evaluate visual clarity during arthroscopic surgery; grades 1 and 3 referred to poor and good visibility, respectively [11,15]. The TXA group showed a significantly greater percentage of grade 3 visual clarity than the control group (*p* = 0.036 in Liu et al. [11], *p* = 0.045 in Takahashi et al. [15]).

### 3.5. VAS 1 Day Postoperational

**Figure 5:**
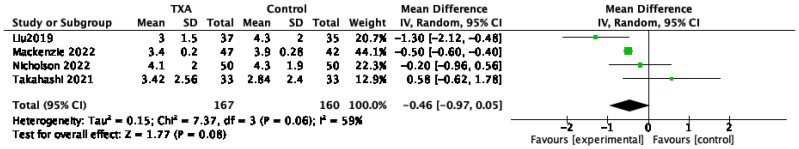
Forest plot and meta-analysis of VAS one day post operation.The centre of each square represents the weighted mean differences for individual trials, and the corresponding horizontal line stands for a 95% confidence interval. The diamonds represent pooled results [11,14,15,20].

Five studies reported visual analog scale (VAS) pain scores on postoperative day 1. The meta-analysis from these five studies demonstrated no significant difference in VAS scores between the TXA and control groups (MD, −0.46 [95% CI, −0.97, 0.05]; *p* = 0.08; Figure 5). Statistical assessment for heterogeneity found for the VAS scores of the TXA versus control groups was I^2^ = 59% (*p* = 0.06). A random-effect model was used. The large degree of statistical heterogeneity indicates underlying differences among the studies. Although the dosage was different from 1–2 g, all four studies were given intravenously before surgery in the TXA group.

### 3.6. Blood Loss

Three studies estimated blood loss with different calculation methods. Liu et al. [11] collected changes in serum hemoglobin and the concentration of hemoglobin in washed irrigation fluid to calculate the estimated blood loss using the Gross formula. Takahashi et al. [15] estimated blood loss by using the gauze visual analog. Gao et al. [19] compared the hemoglobin value between the two groups before and after the operation on day 1. All of them reported no significant difference between the TXA group and the control group.

### 3.7. Secondary Outcomes

#### Operation Time

**Figure 6:**
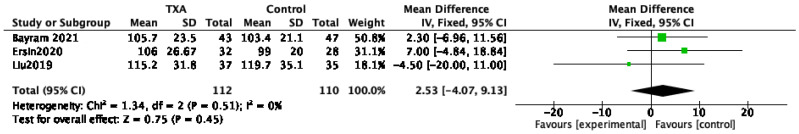
Forest plot and meta-analysis of operation time.The centre of each square represents the weighted mean differences for individual trials, and the corresponding horizontal line stands for a 95% confidence interval. The diamonds represent pooled results [11,12,18].

Five studies recorded the operation time [11,12,15,18,20]. The meta-analysis from these five studies demonstrated no significant difference between the TXA and control groups (MD, −3.51 [95% CI, −15.82, 8.80]; *p* = 0.58). When removing the study by Nicholson et al. [20] for it’s different general anesthesia and epinephrine of irrigation liquid, there was still no significant difference in operation time between the TXA and control groups (MD, 2.53 [−4.07 to 9.13]; *p* = 0.45), and there was less heterogeneity (I^2^ = 0%; *p* = 0.51, Figure 6).

### 3.8. Irrigation Amount Used

**Figure 7:**
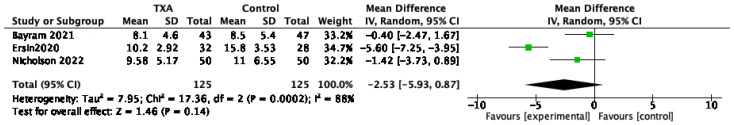
Forest plot and meta-analysis of the irrigation amount used. The centre of each square represents the weighted mean differences for individual trials, and the corresponding horizontal line stands for a 95% confidence interval. The diamonds represent pooled results [12,18,20].

Three studies reported the amount of irrigation solution between the two groups [12,18,20]. The meta-analysis from three studies demonstrated no significant difference between the TXA and control groups (MD, −2.53 [95% CI, −5.93, 0.87]; *p* = 0.14; Figure 7). A random-effect model was used for substantial heterogeneity (I^2^ = 88%, *p* = 0.0002). The large degree of statistical heterogeneity indicates underlying differences among the studies. Having compared the image quality scores of the two groups in three studies, the image quality scores in Ersin et al.’s [12] study were significantly higher in the TXA group than in the controls. Two other studies found similar results for this index [18,20]. However, such a difference in Ersin et al.’s studies was not found to be statistically significant in the subsequent scoring.

### 3.9. Postoperative Shoulder Swelling

Two of the seven included studies assessed postoperative shoulder swelling [11,19]. There were no significant differences in Liu et al. [11], but there were significant differences in Gao et al.’s [19] report between groups at one day post operation. Similarly, the difference disappeared one day post operation. When directly compared, the circumference of the shoulder was measured at two sites: axillary and deltoid or axillary. After the comparison, 1 g TXA (20 mL saline) was given intravenously before the operation and injected into the shoulder joint cavity.

### 3.10. The Need for Pressure Increase and MAP

Two [12,20] of the seven included studies recorded the need for an adjustment in pressure for bleeding. There were no significant differences reported between groups. Two of the seven included studies recorded MAP during the operation [18,20]. There was no significant difference between the control group and TXA group in MAP.

### 3.11. Adverse and Functional Outcomes

Four of the seven studies reported adverse and additional outcomes after the operation [11,14,18,19]. No wound complications or infections or cardiac, thrombotic, or thromboembolic complications were recorded in either group. Six cases in the TXA group and two cases in the control group experienced subcutaneous bloody ecchymosis in Gao et al.’s study [19]. Five of six cases of frozen shoulder occurred in the control group and one in the TXA group; one of three patients with re-tears underwent reverse shoulder arthroplasty, as reported by Mackenzie et al. [14]. Functional outcomes were assessed by Mackenzie et al. [14], and there was no difference between groups in [2,8,24], and 52 weeks in the American Shoulder and Elbow Surgeons Standardized Shoulder Assessment Form or Constant score.

## 4. Discussion

Across the 537 patients in the included studies that were administered TXA, there was no increased incidence of VTE in any study group. Seventeen of them experienced adverse effects owing to the operation and stage of the disease rather than TXA. However, currently, compared with the placebo, there is no evidence to support that the use of TXA before arthroscopic RCR procedures obviously clears the visual field and reduces blood or pain release.

Visualization during arthroscopy depends on several factors, namely: blood loss, which is influenced by arthroscopic pump pressure, and MAP during the operation. Negative results regarding the visual field in this meta-analysis should be explained cautiously. Bayram proved that adding TXA to the irrigation fluid during arthroscopic RCR provides visual quality similar to that of epinephrine, which has been certified to decrease blood loss and improve arthroscopic visualization [4,31,32]. Cenatiempoa et al. [13] reported significantly lower bleeding during surgery (*p* = 0.018) in the TXA group in the public abstract. Five studies assessed the visual field in the operation in different manners [11,12,15,18,20]. Although three of these values were significantly better in the tranexamic acid group, the difference was quite small [11,12,15]. Takahashi et al. [15] reported that the percentages of grade 1 and 2 visual clarity in the TXA group were similar to those in the placebo group (*p* = 0.6 and 0.064, respectively). Ersin et al.’s trial [12] showed that the overall image quality scores in the arthroscopic operations were significantly higher in the TXA group than in the controls. However, the image quality of the intraoperative individual stages, including intraarticular diagnostic arthroscopy, biceps tenotomy, subacromial bursectomy, acromioplasty, tear imaging, anchor placement, suture management, knot tying, and lateral row repair, was not different between the two groups. According to Liu et al.’s study [11], the average visual score in the TXA group was slightly higher than that of the control group (*p* = 0.048). In addition, secondary evidence was that there was no statistically significant between-group difference in the estimated blood loss calculated using the Gross formula in Liu et al.’s study (*p* = 0.310) and gauze visual analog in Ersin et al.’s study [12]. These differences may be explained by the variability with regard to the use of epinephrine in irrigation, the dosage or administration of TXA, and/or the scale used to assess visibility.

In addition to blood loss prevention, TXA has been reported to limit postoperative pain by preventing excessive hematoma formation. However, the results of this systematic review provided different opinions, and there was no significant difference in VAS scores between the two groups. In addition, the anesthesia measurements were obviously different between them: general anesthesia combined with an echo-guided interscalene plexus block in two studies, only general anesthesia in Takahashi et al.’s study [15], and a preoperative interscalene brachial plexus nerve block in Nicholson et al. [20]. Only Takahashi et al.’s study [15] described the postoperative pain control method. Meanwhile, most of the other secondary outcomes (irrigation amount used, postoperative shoulder swelling, the need for pressure increase, MAP, and operative time) were not significantly different between the groups, so TXA could not be analyzed to reduce the VAS scores. As an antifibrinolytic agent, TXA has been shown to be effective in reducing intraoperative bleeding and attenuating the inflammatory response [33]. The difference in visualization and blood loss might be more distinct by conducting large sample RCTs and increasing the dosage or administration combination of drugs.

TXA applied in open shoulder surgery and knee arthroscopic surgery has been found to be effective in reducing blood loss, which directly influences visualization in shoulder arthroscopic operations [10,34]. However, there was dissonance compared with this systematic review and meta-analysis. The blood loss of tranexamic acid used in the ACL reconstruction cases and shoulder surgery was assessed via drain output at 24 h. Only two studies related to shoulder arthroscopy analyzed blood loss [11,15]. In addition, the analysis included two trials considering the change in hemoglobin from the preoperative level to the day 1 postoperative level and revealed no difference between the TXA group and the control group (*p* = 0.07). The statistically significant result excluded the two studies involving arthroscopic procedures. Unlike that in shoulder arthroplasty and ACLR (anterior cruciate ligament reconstruction), the blood loss of shoulder arthroscopic surgery was very low and computed little statistical analysis in this meta-analysis. Blood loss was estimated by the Gross formula or gauze visual analog rather than the drain output. The frequency, dosage, and administration were also obviously increased in shoulder arthroplasty.

The most worrisome complication of the application of TXA is an increased risk of venous thromboembolic complications. No adverse effects or complications related to TXA were collected in this meta-analysis. This is similar to the meta-analysis of TXA in knee arthroscopic surgery, which found no significant difference in deep vein thrombosis (DVT), pulmonary embolus, or infection rate between the experimental and control groups [10]. In addition, there were no thromboembolic complications recorded in the TXA group in shoulder surgery. Meanwhile, TXA has been frequently used in the context of major orthopedic surgeries, such as total knee arthroplasty, total hip arthroplasty, and spine operations, and complications associated with these procedures should be noted [35]. Frozen shoulder after the operation may be related to postoperative rehabilitation and pain control [36]. Arthroscopic capsular release can successfully restore range of motion [37]. Rotator cuff tear size (tear dimensions, tear size area, and tear thickness) showed stronger associations with re-tears at 6 months after surgery [21].

This is an unwonted meta-analysis and systematic review related to shoulder arthroscopic surgery, especially for arthroscopic RCR. The level of evidence and scores of the Jadad Scale showed relatively high-quality RCTs involved. Regardless of the negative results of clear visual field, blood loss, and pain control, TXA use during shoulder arthroscopic surgery appears to be relatively safe. There is no denying that TXA has had successful application in cardiovascular, digestive, orthopedic, and brain surgery. Further works should focus on appropriate dosing, comparisons of the administration routes, the right opportunity, and minimally invasive operation in their analysis. A systematic review including large RCTs with a low risk of bias and the same scale to measure outcomes will help standardize the application of TXA in shoulder arthroscopic procedures.

### Limitations

The main limitation of this review is related to the quantity of available literature on the subject specific to arthroscopic RCR procedures. In addition, only 537 cases of 7 RCTs without language restrictions were included in this review. Another limitation of this study is that a meta-analysis of all seven studies could not be computed certainly due to the variability of dosages and the administration routes of TXA and different scales being performed in each study. Additionally, nine ongoing trials without accessible outcomes increased the risk of bias.

## 5. Conclusions

The use of intravenous or locally tranexamic acid in shoulder arthroscopic surgery did not increase complications or thromboembolic events. Nonetheless, the general use of tranexamid acid cannot be advised to arthroscopic shoulder surgeons because of its weak evidence for reducing bleeding.

## Figures and Tables

**Table 1:** Characteristics of the included studies.

Included Study, Year	Level of Evidence	Country	Procedure	Group (No.)		Administration	Anesthesia Measured	Postoperative Rehabilitation	Pain Control	Measured Outcomes	Complications	Follow-Up
				TXA	Control							
Liu et al., 2019 [11]	II	China	RCR	1 g (37)	Saline (35)	Intravenously 10 min before surgery	General anesthesia combined with anecho-guided interscalene plexus block	Protected with an arm abduction brace	Postoperative analgesia (tramadol/acetaminophen 37.5 mg/325 mg 4 times daily), morphine 4 mg subcutaneously, or ketorolac 30 mg intramuscularly were given if necessary	Visual clarity, analgesic usage, blood loss, operative time, inpatient duration, associated comorbidities, VAS pain score on postoperative day 1, and postoperative shoulder swelling	No thromboembolic adverse effects, wound complications, or infections noted in either group	12 weeks
Ersin et al., 2020 [12]	II	Turkey	RCR	10 mg/kg (32)	Saline (28)	Intravenously 20 min before surgery	General anesthesia	NM	NM	Visual clarity, operation time, irrigation amount used, and the need for pressure increase	NM	NM
Gao et al., 2020 [19]	II	China	RCR	0.5 g in 10-mL Saline (30)	Saline (30)	Intra-articularly inject in to the shoulder joint and subacromial space	General anesthesia	Fixed with Shoulder arm strap suspension	NM	Hb reduction and shoulder swelling	6 cases in the TXA group and 2 cases in the placebo group occurred subcutaneous bloody ecchymosis	1 week
Takahashi et al., 2021 [15]	I	Japan	RCR	1 g (33)	Saline (33)	Intravenously 10 min before surgery	General anesthesia	Immobilized for 4 weeks, dflexion and relaxation of the muscles on the day after surgery, strengthen exercises of the rotator cuff and scapular stabilizers at 6 weeks. Rehabilitation was performed at least 3 months after surgery. Full return to sports or heavy labor was allowed after 6 months.	1 g acetaminophen intravenously every 6 h for 24 h postoperatively	Visual clarity, VAS pain scores, blood loss, operative time	NM	1 week
Bayram et al., 2021 [18]	I	Turkey	RCR	0.42 mg per 1 L of saline (43)	0.33 mg of epinephrine per 1 L of saline (47)	Add in irrigation fluid	General anesthesia	NM	NM	Visual clarity, total operating time, potential thrombotic or thromboembolic side effects, mean arterial pressure (MAP), and total amount of irrigation fluid used	No cardiac, thrombotic, or thromboembolic complications	NM
Mackenzie et al., 2022 [14]	I	Australia	RCR	2 g (47)	Saline (42)	Preoperative and intravenously before the surgery	General anesthesia with the use of an interscalene nerve block using bupivacaine 0.75%	Immobilized in a sling for 4–6 weeks followed by a standardized physiotherapy-led protocol involving active assisted range of motion exercises from 6 weeks followed by a graduated strengthening regime from 12 weeks	NM	VAS pain score, American Shoulder and Elbow Surgeons Standardized Shoulder Assessment Form, and constant scores and range of motion	6 cases of frozen shoulder, 5 occurred in the placebo group and 1 in the TXA group, one of 3 patients with re-tears underwent reverse shoulder arthroplasty	52 weeks
Nicholson et al., 2022 [20]	II	USA	RCR	1 g (50)	No TXA (50)	Preoperative and intravenously before the surgery	A preoperative interscalene brachial plexus nerve block	NM	NM	Visualization, VAS pain scores, operative time, final pump pressure, number of increases in pump pressure, total amount of irrigation fluid utilized, MAP, blood pressure, and anesthesia medical interventions for blood pressure	NM	NM

RCR, rotator cuff repair; MAP, mean arterial pressure; NM, not mentioned; TXA, tranexamic acid; VAS, visual analog scale.

**Table 2:** Modified Jadad score.

Study	Study Design	Randomization	Concealment of Allocation	Double Blinding	Withdrawal and Dropout	Total
Bayram et al., 2021 [18]	RCT	2	2	2	1	7
Ersin et al., 2020 [12]	RCT	2	1	2	1	6
Gao et al., 2020 [19]	RCT	2	1	1	1	5
Liu et al., 2019 [11]	RCT	2	2	2	1	7
Mackenzie et al., 2022 [14]	RCT	2	2	2	1	7
Nicholson et al., 2022 [20]	RCT	2	1	1	1	5
Takahashi et al., 2021 [15]	RCT	2	2	2	1	7

The modified Jadad score was used to evaluate the quality of articles, and studies achieving a score of 4 points or more were considered to be of high quality. RCT indicates randomized controlled trial.

## Data Availability

Not applicable.
